# Stereotactic aspiration of spontaneous intracerebral hematoma: Case series

**DOI:** 10.1016/j.ijscr.2020.06.008

**Published:** 2020-06-11

**Authors:** Achmad Fahmi, Heri Subianto, Nur Setiawan Suroto, Budi Utomo, Riyanarto Sarno, Agus Turchan, Abdul Hafid Bajamal

**Affiliations:** a Post Graduate Doctoral Program, Faculty of Medicine, Universitas Airlangga, Indonesia; b Department of Neurosurgery, Faculty of Medicine, Universitas Airlangga, Indonesia; c Department of Public Health and Preventive Medicine, Faculty of Medicine, Universitas Airlangga, Indonesia; d Department of Informatics, Institute Teknologi Sepuluh November, Indonesia

**Keywords:** Spontaneous intracerebral hematoma, Stereotactic aspiration, Safety

## Abstract

**Introduction:**

Minimally invasive techniques such as stereotactic aspiration of spontaneous intracerebral hematoma (SICH) evacuation can minimize brain tissue damage due to surgery. We share our experience of adopting stereotactic aspiration of SICH in our center without compromising safety.

**Presentation of cases:**

Three stereotactic aspiration procedures for SICH immediately after 24 h of onset are reported. All cases showed good results. Level of consciousness of all the patient returned to normal. Two patients could carry out routine activities independently. One patient has left hemiparesis.

**Discussion:**

Stereotactic aspiration of SICH has been newly implemented at our center with acceptable results achieved. Patient selection has an important role in determining the choice of technique. The procedures were done without anticoagulant agent. Stereotactic aspiration of SICH is associated with limited brain tissue damage, shorter duration of surgery, reduced length of stay, faster postoperative healing, and better functional improvement.

**Conclusion:**

Stereotactic aspiration of SICH is a minimally invasive defined strategy for hematoma evacuation without compromising safety.

## Highlights

• Stereotactic hematoma evacuation can be performed without anticoagulant agent.
• Stereotactic evacuation of intracerebral hematoma can be adopted in any center.
• Stereotactic evacuation of intracerebral hematoma is a safe procedure.
• Stereotactic surgery can minimize brain injury.

## 1 Introduction

Spontaneous intracerebral hemorrhage (SICH) has a high mortality and morbidity rate. It places a significant economic burden on hospitals and health care services. The incidence of spontaneous supratentorial ICH was 20 cases per 100.000 populations, and more than 70 percent of patients died. Spontaneous ICH causes 10–15 percent of first-ever strokes with a 30-day mortality rate of 35–52 percent, and half of them die within two days after the event [1,2].

Open craniotomy techniques have a mortality rate of 25 percent within three months, and 58.9 percent of patients undergoing surgery have an unfavorable outcome [1]. Currently, minimally invasive techniques such as stereotactic aspiration and endoscopy surgery of ICH evacuation can minimize brain tissue damage due to surgery, shorter duration of surgery, and allow local use of anesthesia. Stereotactic aspiration was our new guideline since 2013. We share our experience stereotactic aspiration of SICH in the center with newly adopted this technique without compromising safety using Leksell stereotactic hematoma evacuator in general anesthesia without any anticoagulant agent. This work has been reported in line with the PROCESS guideline [3].

## 2 Presentation of cases

### 2.1 Case 1

A 45-year-old man presented to the emergency room 30 min after developing left hemiparesis and SICH was diagnosed. He refused the treatment on admission. Seven hours later, the patient was brought back to the hospital with diminished level of consciousness and left hemiparesis. The Glasgow Coma Scale (GCS) was E3V3M5.

Head MRI showed acute ICH in the right basal ganglia with volume 55 cc. The perifocal edema displaced the right lateral and third ventricles, causing midline shifting as much as 5 mm to the other side. The patient consented to undergo stereotactic aspiration surgery without anticoagulant for evacuating the clot.

**Fig. 1:**
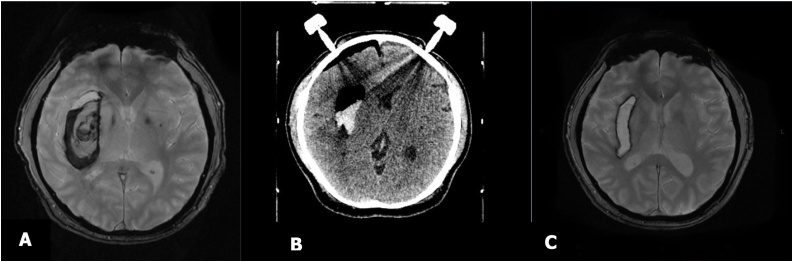
A. MRI image performed a few hour after onset, B. Post-operative CT Scan after stereotactic surgery immediately, and C. MRI image 2 months after surgery.

Stereotactic aspiration of ICH was performed under general anesthesia 10 h after admission. Ten cc of residual hematoma was detected on repeat CT scan and decision was made to manage it conservatively. The patient was treated for 23 days with a satisfactory result. GCS improved to E4V5M6 with residual left hemiparesis. Fig. 1 shows MRI scan two months after surgery.

### 2.2 Case 2

A 52-year-old woman was admitted to the hospital 15 min after developing a decline in level of consciousness. The GCS was E4V2M5 and no hemiparesis. Head MRI showed hyperacute ICH in left temporal lobe (left external capsule and left corona radiate) with volume of 25.6 cc. The perifocal edema pushed the left lateral ventricle, causing midline structure deviation to the right side as much as 6 mm.

**Fig. 2:**
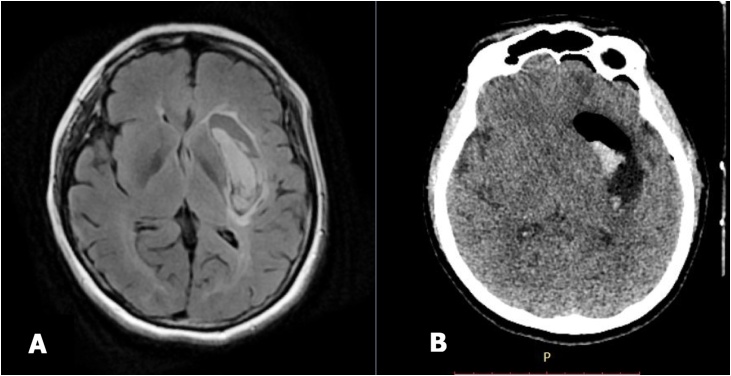
A. Pre-operative MRI a few hour after onset, and B. Post-operative CT scan after stereotactic surgery immediately.

We performed stereotactic surgery without anticoagulant for ICH evacuation 6 h after the onset of symptoms. Fig. 2 presents the postoperative head CT scan showing 80 percent reduction in the amount of hemorrhage. The patient was treated for 11 days. She had GCS E4V5M6 and was able to do routine activities independently before discharge.

### 2.3 Case 3

A 27-year-old girl arrived in the emergency room with diminished consciousness 30 min after the onset of symptoms. The GCS was E3V2M5 with no hemiparesis. A head CT scan revealed ICH on the frontal and parietal regions with a volume of 35 cc causing midline shift to the right side as far as 4 mm. There was non-communicating hydrocephalus, intraventricular hemorrhage, and brain edema.

**Fig. 3:**
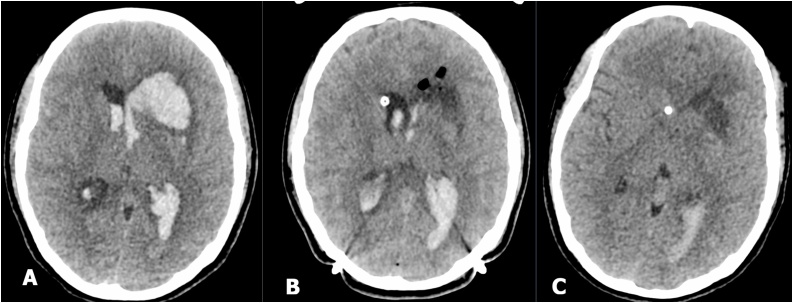
A. Head CT scan a few hour after onset, B. Head CT scan image after stereotactic aspiration immediately, and C. Head CT scan image taken a week after surgery.

Stereotactic aspiration surgery for evacuating the hemorrhage and external ventricular drainage was done without any anticoagulant. The post procedure CT scan showed 90 percent reduction in the amount of hemorrhage. A week later another CT scan was repeated. We found that the ICH in left subcortical frontal lobe (left basal ganglia) had significantly reduced (Fig. 3). The patient was treated for 16 days. Her level of consciousness was E4V5M6 with no neurological deficits, and she was able to take on routine activities independently.

## 3 Discussion

Several studies indicate that patients who receive minimally invasive therapies such as stereotactic aspiration and endoscopic surgery have improved outcomes in the form of minimal tissue damage, less blood loss, reduced brain swelling or edema, reduced operative time, shortened length of stay, faster postoperative healing, and better functional improvement [4].

A meta-analysis study compared stereotactic aspiration with medical therapy for 740 patients with spontaneous ICH. The results showed that mortality due to the ICH was most common in patients who received medication therapy compared with the stereotactic aspiration of hematoma evacuation [1]. Li et al. conducted a research about the comparison between the open craniotomy, stereotactic aspiration, and endoscopic surgery in the management of ICH and found that a number of surgical procedures for evacuating clot such as open craniotomy turned out to be more disadvantageous because of brain tissue damage. They also analyzed and compared the safety and efficacy of stereotactic aspiration, endoscopic surgery, and craniotomy for the treatment of spontaneous supratentorial lobar ICH [4].

According to a meta-analysis of randomized controlled trials comparing stereotactic aspiration versus craniotomy for primary ICH, stereotactic aspiration has several benefits, including: (1) Significantly decreased odds of death or dependency in patients with primary ICH. (2) No significant difference in the total risk of complications between the groups with either stereotactic aspiration or craniotomy treatment. (3) Significantly reduced risk of re-bleeding with stereotactic aspiration in comparison with craniotomy. (4) Significantly reduced risk of death/dependence, death, and GI hemorrhage compared to conventional open craniotomy. (5) Significantly decreased risk of re-bleeding compared to key-hole craniotomy [5].

The prognosis of ICH depends on the location of the bleeding (supratentorial or infratentorial), size of the hematoma, level of consciousness, age, and general condition of the patient. Death after 30 days of ICH ranged from 35 to 52 percent; half of them happened in the first two days of onset [6]. In our cases, the patients experienced a fairly good improvement after performing stereotactic aspiration surgery. Even two of them are able to carry out the physical activity normally without any neurological deficits, while one patient still has weakness in half of the body. This is similar to the research which was conducted by Kim, et al., who examined the functional outcome of spontaneous ICH patients with hematoma volume less than 30 ml and level of consciousness more than 13 who underwent stereotactic aspiration. The results showed that there was an increase in functional outcomes and improvement in functional recovery to perform daily life activities [7].

## 4 Conclusions

Stereotactic aspiration of SICH technique without anticoagulant agent provides surgeons with a defined strategy for hematoma evacuation without compromising safety. Patient’s selection still has an important role in deciding open craniotomy or stereotactic aspiration or other techniques. Stereotactic aspiration of hematoma evacuation can minimize brain tissue damage due to surgery, shortens duration of surgery, reduces length of stay, enhances postoperative healing, and improves functional outcome.

## Declaration of Competing Interest

None.

## Sources of funding

None.

## Ethical approval

All of the procedures performed in this study involving human participants were in accordance with the ethical standards of the institutional research committee.

## Consent

All of the patient had sign informed consent for the surgery.

Patient identity doesn’t seen in this case report.

## Author contribution

Achmad Fahmi, MD, Ph.D: study concept or design, data collection, data analysis or interpretation, writing the paper.

Heri Subianto, MD: study concept or design, writing paper.

Nur Setiawan Suroto, MD: study concept or design.

Budi Utomo, MD, PhD: study concept and critical revised article.

Prof. Riyanarto Sarno: study concept and critical revised article.

Agus Turchan, MD, PhD: study concept, critical revised article and supervising.

Prof. Abdul Hafid Bajamal: study concept, critical revised article and supervising.

## Registration of research studies

Name of the registry: http://www.researchregistry.com.

Unique identifying number or registration ID: researchregistry5506.

Hyperlink to your specific registration (must be publicly accessible and will be checked): N/A.

## Guarantor

Achmad Fahmi, MD, Ph.D

Post Graduate Doctoral Program, Faculty of Medicine, Universitas Airlangga, Indonesia.

## Provenance and peer review

Not commissioned, externally peer-reviewed.
